# The Principles of Physiology Applied to the Preservation of Health, and to the Improvement of Physical and Mental Education

**Published:** 1834-07-01

**Authors:** 


*The Principles of Physiology applied to the Preservation of Health, and to the Improvement of Physical and Mental Education.* By ANDREW COMBE, M.D., Fellow of the Royal College of Physicians of Edinburgh.—Edinburgh, 1834. 8vo. pp. 320.

THIS treatise is upon the whole sensibly written, and, though not altogether free from what might be termed physiological pedantry, is well adapted for non-professional readers. The common fault of hygienic works is their intolerable rigour, —their prophylactic puritanism. A man who lived according to their fantastic notions might immediately be known not to belong to our profession, from his horror of mushrooms, raw water, and sitting on the grass; just as Theophrastus was known to be a foreigner by the Athenian herbwoman, from the very excess of his Atticisms. Dr. Combe, however, does not fall into this error, and is tolerant as long as toleration is a virtue. The following strictures on stays and other absurdities are very judicious, and should be thundered into the ears of schoolmistresses till reform was forced upon them. Every school has its medical attendant, and how is it that these simple truths are unknown? When the doctor is called in to cobble a crooked spine, why does he not tell how crooked spines are manufactured? Is it from gross ignorance, criminal apathy, or mean subservience?

"To an unreflecting person it may seem a very easy and pleasant service to stand for half a day in the attitude of an Apollo or a Gladiator, as a model to a statuary; but, on trying it, he will find, to his astonishment, that stone-breaking or the tread-mill are pastimes in comparison: in the one case, the muscles which preserve the attitude are kept incessantly on the strain; while, in the other, they enjoy that play and variety of motion for which they were destined by nature. We may easily put the fact to the test, by attempting to hold the arm extended at right angles to the body for the short space of ten minutes. He whose muscles, if indeed capable of the exertion, do not feel sore with fatigue at the end of that time, may think himself peculiarly fortunate in being blessed with a powerful constitution.

"The principle just stated explains very obviously the weariness, debility, and injury to health, which invariably follow forced confinement to one position or to one limited variety of movement, as is often witnessed in the education of young females. Alternate contraction and relaxation, or, in other words, exercise of the muscles which support the trunk of the body, are the only means which, according to the Creator's laws, are conducive to muscular development, and by which bodily strength and vigour can be secured. Instead of promoting such exercise, however, the prevailing system of female education places the muscles of the trunk, in particular, under the worst possible circumstances, and renders their exercise nearly impossible. Left to its own weight, the body would fall to the ground, in obedience to the ordinary law of gravitation; in sitting and standing, therefore, as well as in walking, the position is preserved only by active muscular exertion. But if we confine ourselves to one attitude, such as that of sitting erect upon a chair, or, what is still worse, on benches without backs, as is the common practice in schools, it is obvious that we place the muscles which support the spine and trunk in the very disadvantageous position of permanent instead of alternate contraction; which we have seen to be in reality more fatiguing and debilitating to them than severe labour. Girls thus restrained daily for many successive hours invariably suffer, being deprived of the sports and exercise after school-hours which strengthen the muscles of boys, and enable them to withstand the oppression. The muscles being thus enfeebled, they either lean over insensibly to one side, and thus contract curvature of the spine; or, their weakness being perceived, they are forthwith cased in stiffer and stronger stays, that support being sought for in steel and whalebone which Nature intended they should obtain from the bones and muscles of their own bodies. The patient finding the maintenance of an erect carriage (the grand object for which all the suffering is inflicted,) thus rendered more easy, at first welcomes the stays, and, like her teacher, fancies them highly useful. Speedily, however, their effects shew them to be the reverse of beneficial. The same want of varied motion, which was the prime cause of the muscular weakness, is still farther aggravated by the tight pressure of the stays interrupting the play of the muscles, and rendering them in a few months more powerless than ever. In spite, however, of the weariness and mischief which result from it, the same system is persevered in; and, during the short time allotted to that nominal exercise, the formal walk, the body is left almost as motionless as before, and only the legs are called into activity. The natural consequences of this treatment are, debility of the body, curvature of the spine, impaired digestion, and, from the diminished tone of all the animal and vital functions, general ill health: and yet, while we thus set Nature and her laws at defiance, we presume to express surprise at the prevalence of female deformity and disease!

"It would be easy, were it required, to prove that the picture here drawn is not overcharged. A single instance, from a note appended by Dr. Forbes to an excellent treatise on Physical Education, by Dr. Barlow, of Bath, will suffice. After copying the programme of a boarding-school for young ladies, which exhibits only one hour's exercise, consisting of a walk, arm-in-arm, on the high road, and that only when the weather is fine at the particular hour allotted to it, in contrast with nine hours at school or tasks, and three and a half at optional studies or work, Dr. Forbes adds: 'That the practical results of such an astounding regimen are by no means overdrawn in the preceding pages, is sufficiently evinced by the following fact, a fact which, we will venture to say, may be verified by inspection of thousands of boarding-schools in this country. We lately visited, in a large town, a boarding-school containing forty girls; and we learnt on close and accurate inquiry, that there was not one of the girls who had been at the school two years, (and the majority had been as long,) that were not more or less crooked! Our patient was in this predicament; and we could perceive (what all may perceive who meet that most melancholy of all processions, a boarding-school of young ladies in their walk,) that all her companions were pallid, sallow, and listless. We can assert, on the same authority of personal observation, and on an extensive scale, that scarcely a single girl (more especially of the middle classes,) that has been at a boarding-school for two or three years, returns home with unimpaired health; and for the truth of the assertion we may appeal to every candid father whose daughters have been placed in this situation.'"* (P. 109.)

* "Cyclopædia of Practical Medicine, article *Physical Education*; Vol. i. page 693."

